# *Acanthamoeba* Keratitis: A 12-Year Experience Covering a Wide Spectrum of Presentations, Diagnoses, and Outcomes

**DOI:** 10.1155/2013/670242

**Published:** 2013-06-12

**Authors:** Michael A. Page, William D. Mathers

**Affiliations:** ^1^Department of Ophthalmology, University of Minnesota, Minneapolis, MN, USA; ^2^Casey Eye Institute, Oregon Health and Science University, Portland, OR 97201, USA

## Abstract

*Purpose*. To review characteristics of confocal microscopy, clinical presentation, and clinical outcome in 372 cases of *Acanthamoeba* keratitis (AK) from 1999 to 2011. *Methods*. A retrospective case review was performed on 372 cases of AK diagnosed by confocal microscopy (CFM) at a single institution in Portland, Oregon, from 1999 to 2011. A numbered grading system was devised for describing the relative microscopic severity of the AK infections detected. *Results*. “grade 1,” 94 as “grade 2,” 40 as “grade 3,” and 62 as “grade 4.” Peak incidences occurred during 2000–2002 and 2005–2007. Seasonal variation was noted, with a peak during summer months. For the 231 cases with complete records, 64% indicated a history of soft contact lens use. Nine progressed to multiple failed penetrating keratoplasties (PKPs) or enucleation. *Conclusion*. We report an average of 31 new cases of AK per year from 1999 to 2011. This figure equates to 10.3 new cases/1,000,000/year for the Portland metropolitan area. Patients diagnosed with AK exhibited a wide spectrum of clinical and microscopic characteristics. Soft contact lens use remained the single largest risk factor.

## 1. Introduction

*Acanthamoeba* species are ubiquitous free-living organisms that are typically harmless to humans, but in rare instances can cause severe opportunistic infection. First described as a significant cause of corneal disease in 1974 by Naginton et al., *Acanthamoeba* keratitis (AK) is a rare but potentially devastating amoebic infection of the cornea [1]. The pathogenesis of AK involves parasite-mediated cytolysis and phagocytosis of the corneal epithelium and invasion and dissolution of the corneal stroma [2]. The literature has established contact lens wear as the strongest risk factor for development of AK, with contact lens association reported in up to 75%–85% of cases [3].

Previous studies have estimated a prevalence of 1.2 per million adults and 0.2 (United States) to 2 (United Kingdom) per 10,000 soft contact lens wearers per year [4–6]. Parmar et al. suggested that the incidence might be ten times higher [4]. A dramatic rise in the incidence of AK was seen in the 1980s, largely attributed to increased adoption of soft contact lens wear and the use of nonsterile contact lens solutions and homemade saline tablets [5]. Additional outbreaks in the late 1990s and 2000s have been reported in the US and in Europe and have been linked epidemiologically to a number of possible sources, including contaminated municipal water supplies [7], regional flooding [8], and the use of a widely available multipurpose contact lens disinfecting solution [9, 10].

The purpose of this study was to review characteristics of AK patients examined with confocal microscopy (CFM), their clinical presentation, and the clinical outcome on 372 cases of *Acanthamoeba* keratitis diagnosed at a single academic medical center (Casey Eye Institute, Portland, OR, USA) from 1999 to 2010. 

## 2. Methods

**Figure 1:**
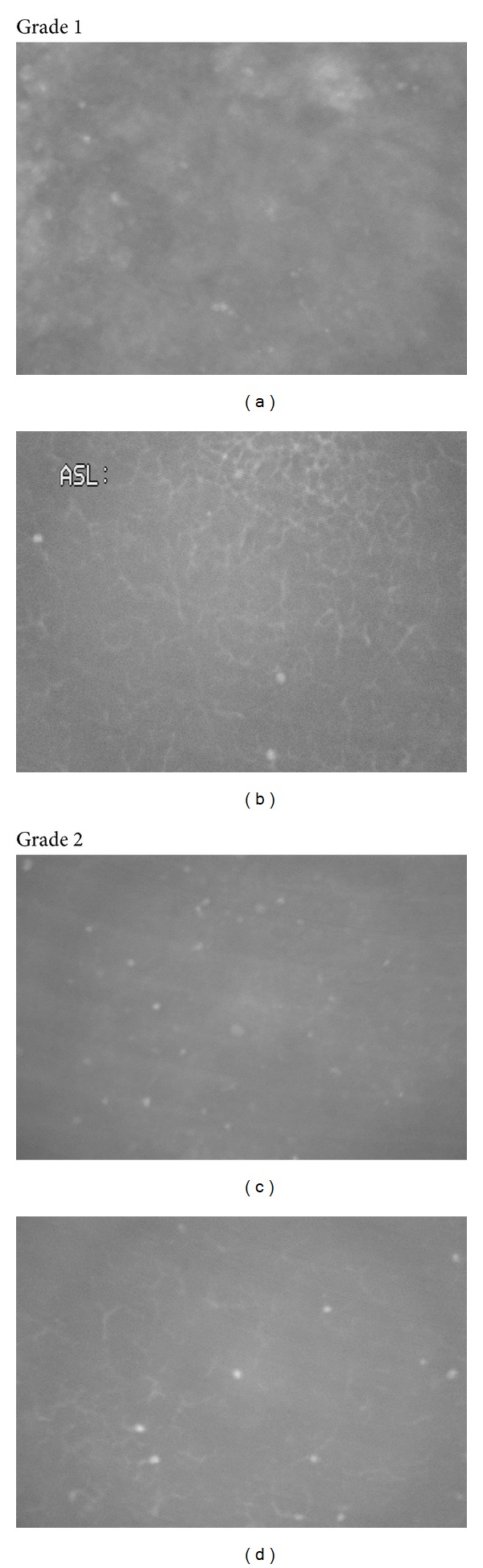
Severity grade 1 ((a) and (b)): few cysts, confined to epithelium (note variable size of *Acanthamoeba* organisms in (a) and Langerhans cells in (b)), confined to small area of the cornea. Severity grade 2 ((c) and (d)): more numerous cysts with clearer morphology, limited to epithelium, confined to small area of the cornea.

**Figure 2:**
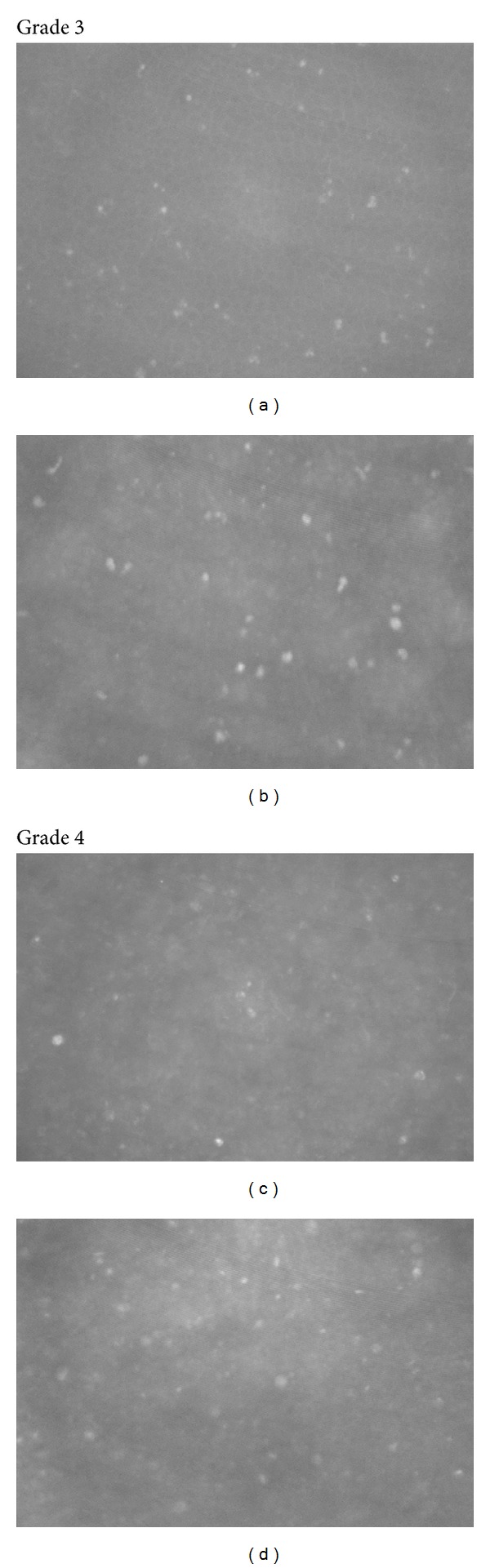
Severity grade 3 ((a) and (b)): numerous cysts, definite morphology, may or may not involve deeper corneal tissues, more densely concentrated. Severity grade 4 ((c) and (d)): numerous cysts and trophozoites, definite morphology, deeper corneal layers involved, densely concentrated, wide corneal area involvement.

A retrospective case review from January 1999 to June 2011 was carried out for 826 consecutive confocal microscopic examinations on patients referred for evaluation of possible *Acanthamoeba* keratitis. All subjects had some form of keratitis by slit lamp examination and some symptoms suggestive of *Acanthamoeba*. We diagnosed 372 cases of AK by CFM that required treatment. The diagnosis was primarily based on digital video images produced by the same confocal microscope (ASL 1000, Model OS-1, Advanced Scanning Inc, New Orleans, LA). A single clinician (WDM) performed and interpreted the confocal microscopic evaluation in each case. Confocal microscopy was considered positive for *Acanthamoeba* if characteristic highly reflective round or ovoid structures with a diameter of 10–25 microns were visualized, or if double-walled structures denoting *Acanthamoeba* cysts were noted. No confocal images suggesting the presence of *Acanthamoeba* were observed in otherwise normal corneas. A numbered grading system was devised to describe the relative microscopic severity (grade 1 = least severe; grade 4 = most severe). See Figures 1 and 2 for representative images of the grading scheme. Medical records were available for review in 231 of the 372 cases. Data on presenting visual acuity, concurrent or previous corneal disease, and soft contact lens use were recorded (Table 1). Of the 231 cases with available medical records, 112 underwent corneal culture for *Acanthamoeba* in addition to the CFM exam; culture results were recorded when available. Outcome characteristics including visual acuity at the time of resolution of microscopic findings were available and recorded for 128/372 cases (Table 2).

## 3. Results

**Figure 3:**
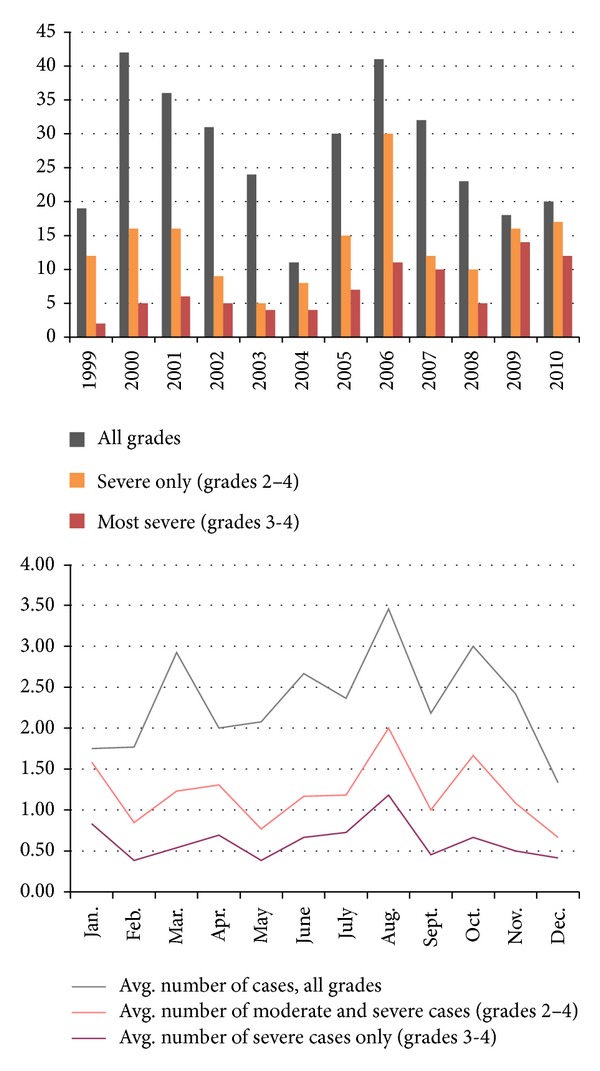
Number of AK cases diagnosed by CFM from 1999 to 2010, annual and monthly average, denoted by grade.

Of the 372 cases, 186 were described as “grade 1 (mild),” 94 as “grade 2,” 40 as “grade 3,” and 62 were “grade 4” the most severe. There were relative peaks in overall incidence noted for the years 2000–2002 (44.3 cases per year) and 2006 (45 cases per year). An upward trend in the incidence of “most severe” cases (grades 3-4) was noted from 2004 to 2010 except for 2008. There was a trend toward seasonal variation with more severe cases and more cases overall occurring during the summer months, peaking in August (Figure 3).

Medical records were available for review in 231 of the 372 cases. The lack of access to medical records in 100% of the cases was due to the fact that over 1/3 of cases were referred for confocal microscope exam only, with all medical management being performed by the outside referring provider. Eighty-one (35%) of the cases with reviewable records presented with Snellen acuity of 20/25 or better in the affected eye, 113 (49%) presented with 20/30 to 20/100 acuity, and 37 (16%) presented with 20/125 or worse acuity. Thirty-nine (17%) of the cases had a history of Herpes simplex (HSV) infection (prior or concurrent, diagnosed clinically by the presence of dendritic or disciform keratitis or by response to topical antiviral medication). One hundred and forty-eight of the 231 records (64%) indicated a history of soft contact lens use. One hundred and twelve cases were cultured and 32 were found to be positive for AK (28%), whereas 80 (72%) were negative. An additional 119 had culture results that were unknown or culture that was not performed (also likely due to the referral pattern as described in Table 1).

 Apart from HSV infection, 47 of 231 (20%) carried a concurrent medical diagnosis, including recent trauma, diabetic eye disease, atopy, basement membrane dystrophy, epidemic keratoconjunctivitis (EKC), previous refractive surgery, crystalline keratopathy, dry eye, or rosacea keratoconjunctivitis (Table 1).

Many cases were lost to followup after the microscopic diagnosis was made, usually when referring providers resumed management. One hundred and twenty-eight cases were followed and managed in our clinic through resolution of amoebic infection based on resolution of CFM findings. Eighty-nine (69%) of these demonstrated 20/25 acuity or better at the time of AK resolution, 28 (22%) demonstrated 20/30 to 20/100 acuity, and 11 (9%) demonstrated 20/200 or worse acuity. Nine (7%) of these progressed to multiple failed penetrating keratoplasty (PKP) and/or enucleation (Table 2). 

## 4. Discussion

Diagnostically, *Acanthamoeba* keratitis remains one of the more challenging clinical entities in corneal disease. Classically the infection produces a ring infiltrate and exquisitely painful radial perineuritis [11], but in reality AK can present with a wide variety of clinical findings and objective confirmation of the diagnosis can be elusive. Culture provides inconsistent results, typically requiring prolonged incubation times (especially if amoebicidal antibiotics have been used in treatment), with published sensitivities ranging from 7% to 52% [12, 13]. Cytopathological methods have been shown to be somewhat more reliable, but they are also relatively invasive [14]. In 2008, Tu et al. compared in vivo tandem-scanning confocal microscopy (CFM) to superficial corneal smear and superficial corneal culture and analyzed CFM for validity against a microbiologic standard. They demonstrated that CFM provided a sensitivity of 90.6% and specificity of 100.0% across 53 patients with both objective and clinical characteristics of AK, whereas smear techniques were positive in 30/41 cases (73% sensitivity) and culture positive in 23/42 cases (52% sensitivity) [13]. Other authors have corroborated the high levels of sensitivity (94%–100%) and specificity (84%–100%) of CFM for amoebic infection [4, 15], but the technology tends to be expensive, not widely available, and moderately operator dependent. Polymerase-chain-reaction- (PCR-) based diagnostics may be an excellent adjunct but have not yet been standardized or become widely available. The authors previously showed that 77% of eyes diagnosed as AK by CFM revealed evidence of *Acanthamoeba* using available PCR techniques [12, 16, 17]. 

This referral center reports an average incidence of 31 new cases of AK per year over the last 12 years with the majority of diagnoses made by confocal microscopy. With a catchment area of approximately 3 million people in the Portland metropolitan area and surrounding communities, this figure equates to 10.3 new cases per million population per year. Even when the CFM grade 1 infections are removed from consideration, the incidence of more severe grade 2 to grade 4 infections remains 4.6 per million per year population, which is substantially higher than usually reported [5]. 

Sixty-four percent of cases in this study were contact lens wearers. Since approximately ten percent of our 3 million catchment population wore contacts, we calculated the incidence of AK to be 0.66 per 10,000 contact lens wearers per year. Counting only the most severe cases (grades 3 to grade 4), we diagnosed 8.5 cases of AK per year, or 0.18 per 10,000 contact lens wearers per year, similar to the reported national average for the United States. 

The national trends in AK incidence as noted by the Centers for Disease Control in a recent publication [10] are reflected in the data presented here. These trends include (1) an overall rise in cases starting in 2004 and (2) a spike in incidence from 2005 to 2007 (note the peak in new cases, 2006, Figure 3). In 2007, the CDC released a preliminary report regarding a perceived outbreak of AK over the previous 2 years, using data drawn from 13 centers nationwide [9]. These findings prompted a larger-scale investigation across 30 states published in 2009. In the case-control study published by the CDC, multivariate risk factor analysis demonstrated a 17-fold risk of AK among contact lens wearers who used a particular multipurpose cleaning and disinfection solution (AMO Complete Moisture Plus). Unopened stock of the solution was tested for *Acanthamoeba* and not found intrinsically contaminated. The product was voluntarily recalled from the market by the manufacturer, and the CDC's conclusion was that the contact lens solution's anti-*Acanthamoeba* efficacy was likely insufficient [10]. It is not known how many patients evaluated at this institution may have used the AMO Complete Moisture Plus Solution.

The seasonal variation in AK incidence, with higher numbers occurring during summer, is well demonstrated in Figure 3 and has been corroborated in a recent paper from Canada [18]. In their analysis of 45 cases of AK from 1999 to 2006, McAllum et al. also noted an overall increase in annual incidence since 2004 and a statistically significant trend toward summer onset. 

Though the data presented here reflects AK trends in the US and Canada as mentioned earlier, there will be questions regarding the relatively high incidence of AK reported in this retrospective series. We propose several possible explanations.

Local environmental factors may play a role. The reported incidence of AK is approximately 15 times higher in the United Kingdom than in the US [6, 7, 19]. The predominant theory to explain this disparity relates to higher levels of amoebae in municipal water supplies and the widespread use of rooftop cisterns in UK communities. In 2004, Kilvington et al. sampled fresh water tap outlets from the homes of 27 patients with confirmed AK; free-living amoebae were found in 24/27 (89%). *Acanthamoeba* species known to cause keratitis were found in 8/27 (30%). Twenty-four out of the 27 households used rooftop cisterns [7]. Even though rooftop cisterns or analogous systems for household plumbing are not widely in place in the Pacific Northwestern US, there may be other relevant environmental factors related to our relatively high rainfall, humidity, wet soil conditions, or other factors. To our knowledge there has been no formal evaluation of *Acanthamoeba* counts in municipal water supplies, domestic plumbing, or soil in Multnomah county or surrounding counties included in our catchment area. A detailed demographic study of our patient cohort was not performed with regards to city or county of residence, work activities, or travel history, but these would potentially be helpful data.

With one observer (WDM) interpreting the CFM images and in most cases not formally blinded to the clinical exam, observer bias is another possible explanation for the higher incidence of AK reported here. However, it should be noted that the majority of video review and assignment of severity grade was performed months to years after the observer had physically examined the patient, and thus was in effect a blinded process. The CFM grading system employed here was not validated against a highly sensitive, specific, and cost-effective microbiologic gold standard because there is not yet one available. PCR shows promise in this role [12, 17], and we would posit that classification of *Acanthamoeba* by speciation, genotyping, or virulence factors would provide most of the information on severity and prognosis.

Although the spectrum of disease we are reporting is wider, the incidence of severe AK reported here is not dramatically higher than the incidence reported by another comparable center. The University of Texas Southwestern, a department of similar size to ours, also primarily used confocal microscopy to identify AK in a series of 56 cases over ten years. This rate of 5.6 per year is not substantially different from our rate for severe (grades 3-4) infections of 8.5 per year [4]. 

The most likely explanation for our higher overall rate is that the actual incidence of AK is truly greater than currently reported and has a wider spectrum of clinical characteristics than typically appreciated. grade 3 and grade 4 type infections, the most important from a public health point of view, were typically associated with worse outcomes including scarring, recurrent amoebic disease, and need for surgery. Grade 2 infections may have had a better course but sometimes progressed to the most severe clinical endpoints despite aggressive therapy or developed a chronic infection which took up to 6–12 months to clear. Grade 1 infections tended to respond readily to topical therapy, and may have included subclinical infections or very early superficial epithelial infections. In most ophthalmic care settings, these cases may be interpreted as an indolent, intermittent, or nonspecific superficial keratitis and may be responsive to mild topical steroids; we would posit that these cases actually represent undiagnosed mild AK. Both host factors and microbiologic characteristics of individual strains (as suggested earlier) probably play a role in the differing phenotypes noted [20, 21].

Soft contact lens wear remains the most important risk factor for the development of AK, and 64% of the cases reported in this series had a documented contact lens history. This figure may be an underestimate based on incomplete access to the full medical record from referring providers. Correlation with herpes simplex keratitis has been previously reported [22, 23], and our data indicates a likewise association, with approximately 17% of AK cases demonstrating a history of HSV ocular disease or active HSV coinfection. The exact relationship between these two pathogenic processes has yet to be elucidated, and some cases of dendritic or disciform keratitis diagnosed clinically as viral infection may actually represent AK masquerading as HSV [24]. Twenty percent of patients diagnosed with AK in this series carried a concurrent corneal diagnosis apart from HSV infection, including recent trauma, diabetic eye disease, atopy, basement membrane dystrophy, epidemic keratoconjunctivitis (EKC), history of refractive surgery, crystalline keratopathy, or dry eye and rosacea keratoconjunctivitis. Trauma (5.2%), basement membrane dystrophy (4.8%), and dry eye with rosacea (5.6%) were the most common and may represent additional risk factors for the development of AK via disruption of the normal epithelial barrier.

Overall, AK likely is a nationally underdiagnosed condition that is modulated by a number of factors including contact-lens wear and habits, contact lens-related products, local environmental conditions, host factors including concurrent corneal disease, and differential pathogenicity of various *Acanthamoeba* strains. Multiple studies have demonstrated the inefficacy of multipurpose contact lens solutions against *Acanthamoeba *[25, 26], and we support the CDC in their recent statement that “premarket standardized testing of contact lens solutions for activity against *Acanthamoeba *spp. is warranted” [10].

Timely confocal microscopy affords an earlier diagnostic confirmation and allows for reliable, noninvasive detection of *Acanthamoeba* in both mild and severe clinical presentations. In most cases of AK the prognosis is guarded, but early detection may be associated with better outcomes [3, 27]. Given the well-tolerated nature of current topical antiamoebic regimens such as dilute chlorhexidine 0.02%–0.06%, empiric treatment of chronic or recurrent keratitis may be indicated when other treatments fail or when AK is suspected, but there is no access to a confocal microscope or reliable *Acanthamoeba* diagnostics.

## Figures and Tables

**Table 1:** Clinical characteristics and concurrent ocular conditions on presentation or referral.

Presentation characteristics (*N* = 231)	*n *	%
Visual acuity on presentation (worse eye)		
20/25 or better	81	35%
20/30 to 20/100	113	49%
20/125 or worse	37	16%
Definite HSV keratitis (prior or concurrent)	39	17%
Probable HSV keratitis (prior or concurrent)	7	3%
History of Soft contact lens use	148	64%
Acanthamoeba culture result		
Positive	32	14%
Negative	80	35%
Unknown	119	52%

Concurrent presenting ocular conditions	*n*	%

Recent trauma	12	5.2%
Diabetic eye disease	7	3.0%
Atopy	4	1.7%
Basement membrane dystrophy	11	4.8%
EKC	3	1.3%
History of refractive surgery	5	2.2%
Crystalline keratopathy	2	0.9%
Dry eye/rosacea	13	5.6%

**Table 2:** Outcome characteristics.

	*n*	%
Number followed to resolution of AK on CFM	128	—
VA 20/25 or better	89	69%
VA 20/30 to 20/100	28	22%
VA 20/200 or worse	11	9%
Number progressed to multiple failed PK or enucleation	9	7%
